# Retinal Pigment Epithelium Ageing: Cellular and Molecular Mechanisms of Long-Term Homeostasis and Age-Related Dysfunction

**DOI:** 10.3390/cells15080725

**Published:** 2026-04-19

**Authors:** Yijing Yang, Pei Liu, Jiangwei Li, Ying Deng, Li Xiao, Qinghua Peng, Jun Peng

**Affiliations:** 1 Faculty of Traditional Chinese Medicine, Hunan University of Chinese Medicine, Changsha 410208, China; 004761@hnucm.edu.cn (Y.Y.); liupei@stu.hnucm.edu.cn (P.L.); 20222048@stu.hnucm.edu.cn (J.L.); 004762@hnucm.edu.cn (Y.D.); xl_646742655@hnucm.edu.cn (L.X.); pengqinghua@hnucm.edu.cn (Q.P.); 2 Institute of Ophthalmology and Otolaryngology of Chinese Medicine, Changsha 410208, China; 3 Changsha Centre for Innovation in Traditional Chinese Medicine Techniques for the Prevention and Treatment of Retinal Diseases and Visual Function Protection, Changsha 410208, China

**Keywords:** retinal pigment epithelium, ageing, long-lived cells, cellular homeostasis, mitochondrial dysfunction, proteostasis, inflammation, age-related retinal dysfunction

## Abstract

**Highlights:**

**What are the main findings?**

- The retinal pigment epithelium is a valuable model for studying ageing in long-lived post-mitotic cells.
- RPE ageing is shaped by structural, metabolic, proteostatic, and inflammatory dysregulation.
- Age-related change in the RPE is expressed more as functional drift than widespread cell loss.

**What are the implications of the main findings?**

- Loss of coordination among active homeostatic systems is a central feature of RPE ageing.
- Understanding RPE ageing may refine strategies for preserving retinal health in later life.

**Abstract:**

The retinal pigment epithelium (RPE) is a long-lived, highly polarised epithelial monolayer that performs essential functions in retinal homeostasis, including outer blood–retina barrier maintenance, visual cycle activity, metabolic exchange, phagocytic clearance of photoreceptor outer segments, and regulation of oxidative and immune balance. Because RPE cells persist for decades under conditions of sustained oxidative, metabolic, and phagocytic stress, this tissue provides a valuable model for examining how long-lived post-mitotic cells preserve function over time and how age-related dysfunction emerges when that balance weakens. Although much of the current literature on RPE ageing has been shaped by age-related macular degeneration (AMD), age-dependent change in the RPE should not be understood solely as a preclinical stage of disease. Rather, the ageing RPE offers a broader framework for studying cellular maintenance under chronic physiological load. In this review, we synthesise current evidence on RPE ageing across four interrelated domains: structural remodelling, mitochondrial and metabolic imbalance, proteostatic and lysosomal burden, and chronic inflammatory dysregulation. Across these processes, ageing in the RPE is expressed less as widespread cell loss than as progressive decline in cellular organisation, buffering capacity, and functional precision. Structural irregularity, altered mitochondrial regulation, incomplete degradative clearance, and persistent low-grade inflammatory signalling together reduce the ability of the RPE to maintain long-term homeostasis and increase vulnerability to age-related retinal dysfunction. We further argue that ageing in the RPE is best understood not as abrupt failure of isolated pathways, but as gradual loss of system coherence among interacting homeostatic systems that remain active while operating under increasing constraint. This view helps integrate diverse cellular and molecular findings and highlights the RPE as an informative model for understanding ageing in long-lived post-mitotic tissues.

## 1. Introduction

Ageing biology has been shaped largely by studies of proliferative tissues and rapidly renewing cell populations, where turnover, replication-associated damage, and replacement offer tractable models of decline [1,2]. These systems have yielded major insights, but they do not fully capture how ageing unfolds in long-lived, post-mitotic cells that persist for decades with minimal renewal. In such tissues, maintenance of function depends less on replacement than on sustained preservation of structural organisation, metabolic balance, and adaptive stress responses [2,3]. Ageing in this context is not simply a problem of loss, but of how long-term homeostasis is maintained under continuous demand.

The retinal pigment epithelium (RPE) is derived embryologically from the outer layer of the optic cup, and this developmental origin helps contextualise its specialised epithelial identity and lifelong persistence within the mature retina. Unlike cell populations that depend on continuous turnover, the RPE must maintain structural integrity and functional competence for decades while remaining exposed to sustained oxidative, metabolic, phagocytic, and inflammatory burden. This persistence is one reason why the RPE is especially informative for ageing research in long-lived post-mitotic cells. As a highly polarised epithelial monolayer positioned between the neural retina and the choroid, the RPE supports visual cycle activity, outer blood–retina barrier integrity, nutrient and metabolite exchange, phagocytic clearance of shed photoreceptor outer segments, and regulation of oxidative and immune balance [4,5]. These functions are performed under persistent physiological stress. The RPE is continuously exposed to high oxygen tension, light-driven oxidative burden, and lifelong handling of lipid- and protein-rich material derived from photoreceptor turnover [6]. The ability of RPE cells to remain viable and functionally engaged for decades implies the existence of layered stabilisation mechanisms that support long-term cellular maintenance.

Current understanding of RPE ageing has been shaped heavily by studies of age-related macular degeneration (AMD). This disease-oriented framework has been highly productive for identifying pathological pathways, yet it may also narrow interpretation. If age-dependent change in the RPE is viewed mainly as a preclinical stage of disease, broader mechanistic questions become less visible. A central issue is how long-lived, non-dividing cells preserve homeostasis over time, and what forms of instability emerge before overt degeneration becomes morphologically obvious [7,8]. From this perspective, AMD is better regarded as one maladaptive outcome of declining RPE resilience rather than as the inevitable expression of physiological ageing.

Beyond its relevance to retinal disease, the RPE can also be viewed as a physiological model for studying ageing in long-lived post-mitotic cells. Unlike rapidly renewing tissues, the RPE must preserve function for decades without meaningful cell replacement while continuously managing oxidative load, phagocytic burden, mitochondrial demand, lysosomal processing, and immune surveillance. This makes it a useful system in which to examine how canonical hallmarks of ageing are expressed under conditions of long-term maintenance rather than turnover. Several of these hallmarks are already well represented in the RPE, including mitochondrial dysfunction, oxidative stress, impaired lysosomal and proteostatic clearance, altered nutrient sensing, and senescence-associated phenotypes. Other dimensions, such as genomic instability and epigenetic dysregulation, are increasingly implicated but remain less fully resolved in this tissue [9,10,11,12]. By contrast, telomere attrition may be conceptually informative as part of the broader ageing framework, yet is less central to the biology of a largely post-mitotic epithelium than in proliferative systems. Framed in this way, the RPE provides an opportunity not only to catalogue age-related changes, but also to test how established ageing concepts should be interpreted when long-term cellular persistence, rather than replication, is the dominant biological constraint.

Several features make the RPE particularly valuable for ageing research. First, RPE cell number is relatively stable across much of the human lifespan, even as measurable changes arise in cell shape, metabolism, and functional output [13,14]. Ageing is therefore expressed less as widespread cell loss than as progressive alteration in organisation and performance of surviving cells. Second, many age-associated changes develop slowly and do not immediately compromise viability, creating an extended intermediate state in which cells remain alive but operate under increasing constraint. Third, ageing is spatially heterogeneous across the tissue, with distinct macular, equatorial, and peripheral trajectories [13]. These regional differences provide a useful framework for examining how local environment, metabolic demand, and photoreceptor architecture shape ageing outcomes within the same epithelial layer.

**Figure 1:**
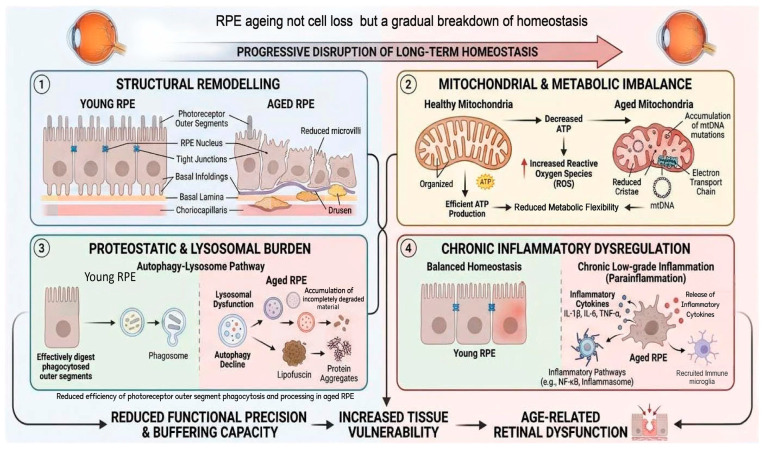
Conceptual framework of retinal pigment epithelium ageing as progressive disruption of long-term homeostasis. The RPE is a long-lived, highly polarised post-mitotic epithelial monolayer that sustains retinal homeostasis under chronic oxidative, metabolic, and phagocytic load. This figure summarises the conceptual framework of RPE ageing developed in this review. Rather than being driven primarily by widespread cell loss or abrupt failure of a single pathway, ageing in the RPE is presented as a progressive disturbance of long-term homeostasis across four interconnected domains: structural remodelling, mitochondrial and metabolic imbalance, proteostatic and lysosomal burden, and chronic inflammatory dysregulation. These interacting changes progressively reduce functional precision and buffering capacity, thereby increasing tissue vulnerability and contributing to age-related retinal dysfunction.

In this review, we examine RPE ageing as a progressive disturbance of long-term homeostasis in a long-lived post-mitotic tissue. We focus on four interrelated domains: structural remodelling, mitochondrial and metabolic imbalance, proteostatic and lysosomal burden, and inflammatory dysregulation. Across these domains, we argue that ageing in the RPE is best understood not as abrupt failure of isolated pathways, but as progressive uncoupling of homeostatic processes among interacting systems that remain active while operating with declining precision and buffering capacity. Framed in this way, the ageing RPE serves not only as a site of retinal vulnerability, but also as a mechanistically informative model for ageing in long-lived cells. As summarised in Figure 1, RPE ageing is best viewed as a progressive disturbance of long-term homeostasis across interacting structural, metabolic, proteostatic, and inflammatory domains.

## 2. Structural Ageing of the Retinal Pigment Epithelium: Loss of Geometric Order Without Overt Cell Loss

One of the earliest and most reproducible manifestations of RPE ageing is alteration in cellular geometry. In youthful tissue, RPE cells form a tightly packed epithelial mosaic with largely hexagonal organisation. This regularity is not merely descriptive. It reflects functional integration among polarity, cytoskeletal tension, adherens junctions, and cell–cell coupling that together support epithelial stability and barrier function.

With age, this spatial order becomes progressively less regular. Morphometric analyses of human RPE have consistently shown increased cell size, greater variability in cell shape, and reduced hexagonal packing, particularly outside the macular region [11,12,13]. Importantly, these geometric changes often occur while overall cell number remains broadly preserved. Structural ageing in the RPE therefore reflects reorganisation of viable cells rather than simple depletion of the epithelial sheet.

This reorganisation is not spatially random. Peripheral RPE cells tend to enlarge and elongate earlier, whereas macular cells often retain more regular morphology until later stages [13,14]. Such regional heterogeneity likely reflects differences in photoreceptor density, metabolic demand, and local mechanical stress. It also indicates that tissue ageing in the RPE is shaped by microenvironmental context rather than by a uniform intrinsic timer.

Changes in geometry are accompanied by remodelling of junctional and cytoskeletal networks. The RPE depends primarily on N-cadherin-based adhesion to maintain epithelial cohesion, with functional coupling to β-catenin and Wnt-related signalling pathways [15]. Experimental oxidative stress disrupts this network, altering junctional protein distribution and actin organisation without immediately abolishing barrier integrity [15,16]. These observations suggest that ageing does not simply eliminate epithelial architecture, but progressively reduces its precision.

In the short term, such remodelling may be adaptive. Changes in adhesion and cytoskeletal tension may allow cells to accommodate increased size or irregular shape while preserving continuity of the monolayer. Over longer periods, however, reduced geometric fidelity is likely to weaken tissue-level alignment. Regions with greater variability in RPE morphometry have been associated with areas later showing heightened vulnerability in ageing and early AMD, even in the absence of marked cell death [12,17]. Structural drift may therefore lower the threshold for dysfunction before overt degeneration occurs.

Extracellular ageing further intensifies these pressures. Thickening of Bruch’s membrane, altered extracellular matrix composition, and accumulation of lipid-rich deposits change both the mechanical and diffusional properties of the RPE–choroid interface [17,18]. These extracellular modifications constrain an epithelial layer that is already adapting to internal reorganisation. Structural ageing of the RPE should therefore be understood as a multilevel process involving both cellular remodelling and progressive change in the matrix environment that supports long-term epithelial stability.

From a physiological perspective, an important unresolved question is how far RPE ageing contributes to visual decline during otherwise healthy ageing. Current evidence suggests that age-related alterations in RPE geometry, metabolic regulation, degradative capacity, and inflammatory tone may reduce retinal resilience and increase susceptibility to dysfunction. However, it remains difficult to isolate the independent contribution of physiological RPE ageing from concurrent changes in photoreceptors, Bruch’s membrane, the choroid, and neural retinal circuitry. For this reason, the contribution of normal RPE ageing to age-related visual decline is best regarded as plausible but not yet definitively quantified.

Related to this issue, available morphometric studies indicate that RPE ageing is often expressed more clearly as altered cell shape, enlargement, irregular packing, and functional drift than as widespread early cell loss. Quantitative analyses in human tissue suggest relative preservation of overall cell number across much of the lifespan, although regional variation and local depletion may become more apparent in vulnerable areas or under disease-associated conditions. This distinction is important because it supports the view that functional vulnerability in the ageing RPE can emerge before overt attrition of the epithelial sheet becomes prominent. Much of the available evidence for morphometric disruption is derived from cross-sectional human tissue analysis, whereas longitudinal human data remain limited.

## 3. Metabolic and Mitochondrial Ageing of the Retinal Pigment Epithelium: Imbalance Rather than Failure

Mitochondrial dysfunction is often presented as a central hallmark of ageing, usually framed as progressive decline in bioenergetic capacity driven by cumulative oxidative damage. In the RPE, however, the picture is more complex. Age-dependent mitochondrial change is evident, but it does not consistently take the form of simple energetic failure. Instead, the ageing RPE more often displays a mismatch between mitochondrial activity, organelle quality control, and cellular demand.

Ultrastructural studies of ageing human RPE reveal increased mitochondrial heterogeneity, including enlarged or elongated mitochondria, altered cristae organisation, and changed subcellular localisation [19,20]. Although such features were initially interpreted as degenerative, comparative work suggests they may also represent long-term remodelling in response to sustained metabolic load. In zebrafish, for example, age-dependent mitochondrial changes in the RPE appear linked to developmental and physiological contexts rather than to straightforward deterioration [21]. This suggests that mitochondrial morphology in ageing tissues may reflect adaptive adjustment as well as damage.

Studies of mitochondrial dynamics support this interpretation. Disruption of PGAM5 impairs DRP1-dependent fission and promotes hyperfused mitochondria with reduced turnover in RPE cells [22]. Importantly, this does not cause immediate ATP depletion. Instead, it is associated with increased ATP, elevated reactive oxygen species, and activation of mTOR- and interferon-related signalling, eventually producing a senescence-like phenotype. These findings argue that mitochondrial ageing in the RPE is not driven primarily by insufficient energy generation, but by weakened alignment between mitochondrial architecture, quality control, and downstream stress signalling.

A similar pattern is seen in stressed or senescence-associated RPE states, where cells often retain or even increase metabolic activity. Transcriptomic and metabolic profiling has shown elevated oxidative phosphorylation, altered substrate use, and increased mitochondrial mass under chronic stress conditions [23,24]. Such findings do not indicate preserved youthful function. Rather, they suggest a compensatory state in which mitochondria remain active while operating under reduced regulatory constraint. Over time, this poorly constrained activity increases oxidative and inflammatory burden.

Nutrient-sensing pathways reinforce this imbalance. In aged RPE, mTORC1 shows increased lysosomal association and enhanced responsiveness to amino acids [25]. This persistent anabolic signalling coincides with impaired degradation of photoreceptor outer segments and reduced autophagic flux. In youthful cells, mitochondrial metabolism, nutrient sensing, and lysosomal degradation are tightly coordinated. During ageing, these relationships become progressively uncoupled, allowing synthetic and signalling activity to persist despite declining clearance capacity.

Mitophagy is also compromised. Evidence from ageing retinal tissue supports reduced efficiency of mitochondrial turnover, leading to retention of organelles that remain metabolically active but are no longer optimally controlled [26,27]. These mitochondria continue to generate ATP and signalling intermediates, yet their persistence amplifies reactive stress and inflammatory tone. In the ageing RPE, mitochondrial dysfunction is therefore best viewed as imbalance rather than exhaustion.

**Figure 2:**
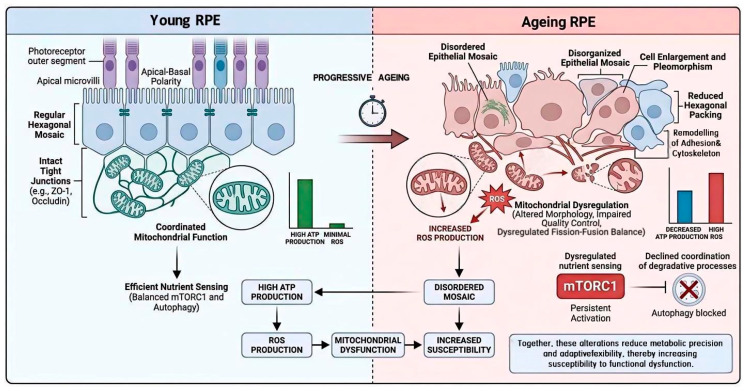
Structural remodelling and mitochondrial–metabolic imbalance in the ageing retinal pigment epithelium. This figure illustrates the coupled structural and metabolic changes that develop during RPE ageing. In youthful tissue, the RPE maintains a regular epithelial mosaic with preserved polarity, junctional organisation, and coordinated mitochondrial function. With age, this spatial and metabolic order becomes progressively destabilised. Structural changes include cell enlargement, increased variability in cell shape, reduced hexagonal packing, and remodelling of adhesion and cytoskeletal networks. In parallel, mitochondria show altered morphology, impaired quality control, dysregulated fission–fusion balance, and persistent reactive oxygen species production. Nutrient-sensing pathways such as mTORC1 remain active in a context of declining degradative orchestration. Together, these alterations reduce metabolic precision and adaptive flexibility, thereby increasing susceptibility to functional dysfunction even before overt structural collapse or cell loss occurs.

Overall, mitochondrial ageing in the RPE exemplifies a broader principle: functional vulnerability can emerge before absolute capacity is lost. Mitochondria remain active, but their outputs become less predictable, less well integrated, and more difficult to buffer. This progressive loss of metabolic balance contributes to age-related dysfunction in a tissue that may still appear structurally intact. The relationship between epithelial remodelling and mitochondrial–metabolic dysregulation is summarised in Figure 2, which highlights how functional vulnerability can emerge before overt tissue loss becomes evident. Interpretation is complicated by the fact that many mechanistic insights derive from in vitro or stress-induced models, which may not fully recapitulate physiological ageing in vivo.

## 4. Proteostasis Collapse as a Chronic Bottleneck in Long-Lived Retinal Pigment Epithelial Cells

Because the RPE is both long-lived and continuously phagocytic, maintenance of proteome integrity imposes an exceptional burden on degradative systems. Unlike proliferative tissues, the RPE cannot dilute damaged proteins and organelles by cell division. It must instead rely on sustained function of lysosomes, autophagy, heterophagy, and the ubiquitin–proteasome system to preserve homeostasis over decades.

One of the most recognisable features of age-related proteostatic stress in the RPE is lipofuscin accumulation. Lipofuscin arises largely from incompletely degraded photoreceptor outer segments and autophagocytosed cellular material, and its abundance increases progressively with age [28,29]. Although components such as A2E possess photoreactive and lysosome-disruptive properties in vitro [30,31], lipofuscin is better understood as a marker of declining degradative efficiency than as a single primary toxin [32]. Its accumulation reflects the fact that the lysosomal system in the ageing RPE remains active, but increasingly operates near saturation.

This narrowing of degradative reserve links proteostasis directly to metabolic ageing. Persistent mitochondrial activity increases the production of damaged proteins and oxidatively modified substrates, while mTORC1 activity remains elevated in a context of declining clearance [25]. In youthful RPE, these systems are balanced. With age, anabolic drive persists while lysosomal and autophagic flexibility declines, creating a mismatch between synthetic load and degradative capacity.

Autophagy and heterophagy are particularly vulnerable to this burden. The RPE must process daily photoreceptor outer segment turnover while also maintaining basal organelle recycling. Age-related dysregulation of autophagy and heterophagy has been strongly linked to RPE dysfunction and AMD susceptibility [27,33]. However, degradation does not stop; rather, capacity becomes progressively constrained. Under these conditions, even modest fluctuations in oxidative or metabolic stress may exceed available reserve.

The ubiquitin–proteasome system contributes to this bottleneck. Age-dependent reductions in proteasomal activity and accumulation of polyubiquitinated proteins have been described in retinal tissues [34,35]. As proteasomal efficiency declines, greater burden is redirected toward lysosomal pathways that are already stressed by lifelong heterophagy. This creates a reinforcing loop of overload.

Post-translational control also influences how proteostatic stress is managed. Altered SUMOylation has been reported in oxidatively stressed and senescence-associated RPE cells, along with changes in inflammatory gene expression and cellular morphology [36]. Inhibition of SUMOylation can attenuate senescence-related phenotypes without removing upstream stress, suggesting that such modifications shape cellular response to proteostatic strain rather than serving as initiating lesions.

**Figure 3:**
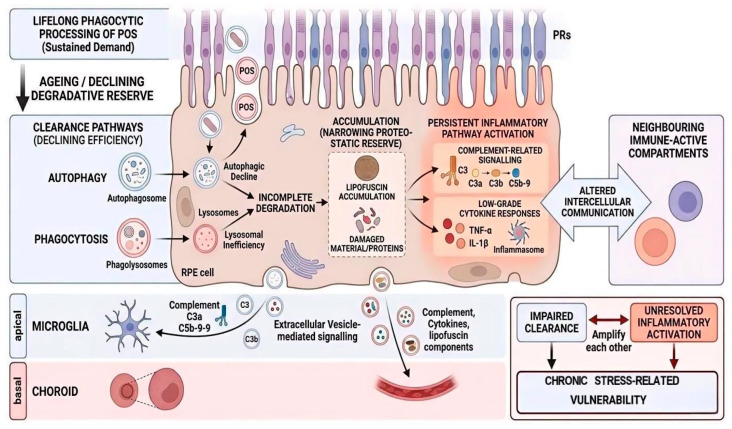
Proteostatic and lysosomal burden drive inflammatory dysregulation and intercellular stress signalling in the ageing retinal pigment epithelium. This figure summarises how declining degradative reserve contributes to inflammatory dysregulation in the ageing RPE. Lifelong phagocytic processing of photoreceptor outer segments places sustained demand on lysosomes, autophagy, and related clearance pathways. With age, incomplete degradation promotes accumulation of lipofuscin and other damaged material, while autophagic and lysosomal efficiency progressively declines. This narrowing of proteostatic reserve is accompanied by persistent activation of inflammatory pathways, including complement-related signalling and low-grade cytokine responses. The ageing RPE also engages in altered intercellular communication with neighbouring immune-active compartments, including microglia and the choroid, in part through extracellular vesicle-mediated signalling. Together, impaired clearance and unresolved inflammatory activation amplify each other, shifting the tissue from adaptive maintenance toward chronic stress-associated vulnerability. POS, photoreceptor outer segments; PRs, photoreceptors.

At more advanced stages, chronic lysosomal stress may destabilise membranes and trigger regulated cell death pathways. Lipofuscin accumulation has been linked to lysosomal membrane permeabilisation and atypical necroptosis in RPE cells [37]. Yet even here, the key point is not abrupt collapse. For extended periods, RPE cells remain viable and metabolically active despite operating with restricted degradative margins. Proteostasis decline in the ageing RPE is thus best understood as progressive loss of buffering capacity. Dysfunction emerges when the system can no longer absorb incremental stress, not when degradation ceases entirely. As shown in Figure 3, proteostatic decline and inflammatory dysregulation should be viewed as mechanistically linked processes that reinforce stress accumulation in the ageing RPE. Evidence for impaired degradative reserve is strong across experimental systems, but the relative contribution of lysosomal, autophagic, and proteasomal decline in human ageing RPE remains incompletely resolved.

## 5. Adaptive Stabilisation During Ageing: Oxidative Defence, Inflammation, and Intercellular Communication

Inflammatory signalling is an intrinsic component of RPE homeostasis. The RPE is neither immunologically inert nor passively exposed to immune processes. It actively regulates complement activity, cytokine tone, antigen presentation, and communication with neighbouring immune-active compartments such as the choroid [7,38,39,40]. In youthful tissue, these functions support maintenance, clearance, and surveillance. During ageing, however, this balance becomes progressively harder to sustain.

Inflammatory change in the ageing RPE should not be understood as an isolated process. Rather, it emerges in the context of structural drift, mitochondrial imbalance, and proteostatic burden. As these systems become more constrained, inflammatory signalling is engaged more frequently and resolved less effectively. In this sense, chronic inflammation in the ageing RPE reflects persistent activation of a homeostatic tool in a setting where the underlying sources of stress are no longer fully contained.

Complement signalling is central to this process. Age-related increases in complement components and inflammatory mediators have been documented in RPE cells and surrounding tissues, with strong links to AMD-associated biology [41,42]. Yet inflammatory activation alone does not define pathology. Human tissue data suggest that inflammatory markers may rise before overt degeneration and may initially represent adaptive responses to impaired clearance [41,42,43]. Problems arise when these responses become persistent rather than transient.

Repeated inflammatory activation changes the character of the response. Cytokine and complement signalling become sustained, while resolution pathways lose efficiency [43,44]. In the RPE, this resembles localised inflammaging, but with tighter coupling to cell-intrinsic stress than to systemic immune ageing [45]. Notably, this state does not necessarily induce immediate cell death. RPE cells may remain viable while inflammatory tone reshapes transcriptional priorities, stress thresholds, and metabolic behaviour.

Intercellular communication with retinal immune cells further shapes ageing trajectories. Microglia respond to stress-associated signals derived from the RPE and in turn modulate the local inflammatory environment [44,46]. Extracellular vesicles, including exosomes, provide one mechanism for this dialogue. RPE-derived vesicles carry proteins, lipids, and microRNAs capable of influencing inflammatory and metabolic states in recipient cells [47,48]. With age, both vesicle abundance and cargo composition shift, increasingly enriching stress- and inflammation-related signals.

These observations highlight a central feature of RPE ageing: the problem is not inflammation alone, but declining systems coherence between inflammatory outputs and the tissue’s capacity for repair and clearance. In young tissue, inflammatory cues are matched to effective recovery. In aged tissue, similar signals persist in a context where metabolic flexibility and degradative reserve are reduced [43,45]. Immune activity then becomes a modifier of vulnerability rather than a reliable mechanism of restoration.

This has important therapeutic implications. Broad suppression of inflammation may interfere with adaptive maintenance responses, whereas unchecked chronic activation amplifies stress [49]. Restoring coordinated signalling, rather than simply suppressing immune pathways, may therefore be the more relevant translational goal. Here again, much of the mechanistic literature is based on experimental models, whereas direct human evidence is often associative rather than causal.

## 6. Theoretical Implications

The ageing RPE offers more than an ocular example of chronic stress adaptation. It provides a mechanistically informative model for studying ageing in long-lived post-mitotic cells. Several established ageing frameworks are visible in this tissue, including mitochondrial dysfunction, proteostasis decline, inflammaging, and senescence-associated states [50,51,52]. However, the RPE also reveals important limits of interpreting these processes through pathway-centric models alone.

Our framework is intended to complement, rather than replace, established ageing models. Several recognised hallmarks of ageing are clearly represented in the RPE, including mitochondrial dysfunction, altered nutrient sensing, impaired proteostasis, lysosomal stress, chronic inflammatory activation, and senescence-associated phenotype. Yet the ageing RPE also illustrates an important interpretive point: in long-lived post-mitotic cells, these hallmarks do not necessarily appear as isolated failures, nor do they progress in a simple linear sequence. Instead, they remain functionally interconnected, and vulnerability emerges as their connectivity weakens over time. In this sense, the present framework extends descriptive integration by emphasising the systems-level relationship among still-active homeostatic processes. This perspective is also compatible with network-based views of ageing, which interpret decline less as failure of a single node than as progressive destabilisation of interdependent regulatory networks. In the RPE, such a view helps explain why functional drift can become evident before overt cell loss or gross structural collapse.

Mitochondrial ageing in the RPE is not defined simply by energetic loss. Mitochondria often remain active, and sometimes hyperactive, even as quality control deteriorates. This suggests that declining regulatory fidelity may be more informative than declining capacity alone [19,20,24,25]. Proteostasis decline unfolds as progressive loss of elasticity rather than threshold collapse. Clearance systems continue to function, but with narrowing reserve [33,34,53]. Inflammaging in the RPE reflects repeated activation of compensatory pathways in a context of declining resolution, rather than unidirectional immune excess [38,43,45,54].

The RPE is also useful because it places classical ageing hallmarks into a tissue context dominated by cellular persistence rather than turnover. Mitochondrial dysfunction, oxidative stress, lysosomal impairment, proteostatic burden, and dysregulated mTOR signalling are all strongly represented in the ageing RPE and can be tracked in relation to functional decline. By contrast, genomic instability and epigenetic dysregulation are increasingly recognised as relevant but remain less completely integrated into current models of physiological RPE ageing. Their contribution may be especially important in linking chronic metabolic and redox stress to durable changes in transcriptional state. Telomere attrition, although central to many ageing frameworks, is more difficult to interpret in a tissue that is largely post-mitotic under physiological conditions. For this reason, the ageing RPE does not simply reproduce the canonical hallmarks of ageing; it helps distinguish which of them remain most informative in long-lived non-dividing cells.

Recent high-resolution profiling approaches are beginning to add further resolution to this framework. For example, single-cell profiling has revealed previously underappreciated heterogeneity within adult human RPE populations, while transcriptomic analyses of aged mouse RPE support age-associated upregulation of inflammatory, immunogenic, and oxidative stress programmes [55,56]. Spatial transcriptomic studies in retinal stress models further suggest that ageing-related vulnerability may be regionally patterned rather than uniformly distributed [57]. These approaches do not yet resolve the full temporal architecture of RPE ageing, but they strengthen the view that age-related change is heterogeneous, state-dependent, and spatially organised rather than uniform across the tissue. A concise summary of the principal structural, molecular, and functional changes reported in the ageing RPE is provided in Table 1.

Senescence poses an additional conceptual challenge. Senescence-associated markers, including p16^INK4a^, p21^CIP1^, and SA-β-gal activity, have been described in ageing or stressed RPE cells [60,61]. Yet the RPE is post-mitotic, and these phenotypes cannot be mapped straightforwardly onto the classic model of irreversible cell-cycle arrest. In this tissue, senescence-like states may overlap with adaptive stress programmes rather than representing uniform terminal dysfunction. This complicates direct application of senolytic logic to the ageing RPE and highlights the need for more precise definitions of senescence in long-lived non-dividing cells.

For clarity, senescence-like state in the RPE is used here in an operational rather than strictly classical sense. We use the term to describe cells that remain viable but exhibit persistent stress signalling, altered inflammatory tone, metabolic reprogramming, reduced degradative flexibility, and expression of senescence-associated markers such as p16, p21, or SA-β-gal activity. This state should be distinguished from stress adaptation, in which compensatory responses remain sufficiently coordinated to preserve function, and from overt dysfunction, in which homeostatic precision declines without necessarily fulfilling a senescence-associated phenotype. Because the RPE is post-mitotic, these categories may overlap, but distinguishing them helps avoid treating all chronic stress-associated change as equivalent to classical proliferative senescence.

One example that illustrates how the RPE may serve as an experimentally tractable system for mitigating senescence-associated stress responses is NAD^+ metabolism. Age-dependent reduction in NAD^+ availability has been linked to impaired metabolic resilience and cellular senescence in the RPE, and loss of NAMPT in ageing RPE has been reported to reduce NAD^+ levels while promoting senescence-related phenotypes [58]. In a complementary injury-based model, nicotinamide mononucleotide supplementation attenuated sodium-iodate-induced senescence and inflammatory responses in RPE cells [59]. Although these findings do not establish NAD^+ restoration as a validated therapy for physiological RPE ageing, they provide a concrete RPE-relevant example of how mitochondrial-metabolic support may modify stress adaptation, inflammatory tone, and senescence-like phenotypes in a long-lived epithelial tissue. More broadly, such interventions are conceptually important because they target regulatory resilience rather than a single downstream lesion, which is consistent with the view advanced here that ageing in the RPE reflects progressive loss of coordination among still-active homeostatic systems.

A related implication concerns model choice. If the RPE is to be used as a general model of ageing biology, the strengths and limitations of available experimental systems must be made explicit. Immortalised lines such as ARPE-19 and hTERT RPE-1 offer accessibility, reproducibility, and suitability for mechanistic perturbation, but they do not fully reproduce the mature phenotype, polarity, and cumulative lifetime burden of native human RPE. Induced pluripotent stem cell-derived RPE provides a valuable human platform with greater differentiation relevance and disease-modelling flexibility, yet it likewise lacks the decades-long exposure that defines physiological ageing in vivo. Primary human RPE remains highly informative because it preserves closer tissue identity, but it is constrained by donor availability, variability, and limited lifespan in culture. Rodent material offers experimental depth and temporal control, but introduces a major anatomical limitation: rodents lack a true macula and therefore cannot fully model the regional specialisation and macula-centred vulnerability that shape human RPE ageing and AMD-associated change. Progress will therefore depend less on identifying an ideal single model than on integrating complementary systems according to the specific hallmarks or mechanisms under study.

Important questions remain, but the main challenge is no longer a lack of descriptive detail. Rather, it is the need to define the temporal and causal architecture of RPE ageing with greater precision. Longitudinal approaches capable of tracking structural, metabolic, proteostatic, and inflammatory trajectories within the same tissue will be essential for clarifying how dysfunction develops over time. Causality among established ageing markers also remains uncertain. Lipofuscin accumulation, mitochondrial remodelling, altered nutrient sensing, and inflammatory activation are well described, yet their causal relationships remain incompletely resolved. In many cases, these changes may be consequences of declining buffering capacity rather than primary initiating lesions. Experimental designs that perturb one process while preserving others, and that capture system-level responses rather than isolated readouts, will be necessary to distinguish cause from consequence more rigorously.

An integration-based view of RPE ageing also has translational implications. If dysfunction arises less from isolated pathway failure than from progressive uncoupling among mitochondrial regulation, nutrient sensing, proteostatic reserve, lysosomal clearance, and inflammatory control, then therapeutic strategies may need to target restoration of regulatory resilience rather than a single downstream lesion. In this context, pathways related to mTOR signalling, mitophagy, autophagic–lysosomal capacity, and proteostasis become especially relevant. Such an approach may also refine how age-related macular degeneration is conceptualised therapeutically, by shifting attention from end-stage manifestations toward preservation of long-term homeostatic coherence in the ageing RPE.

**Figure 4:**
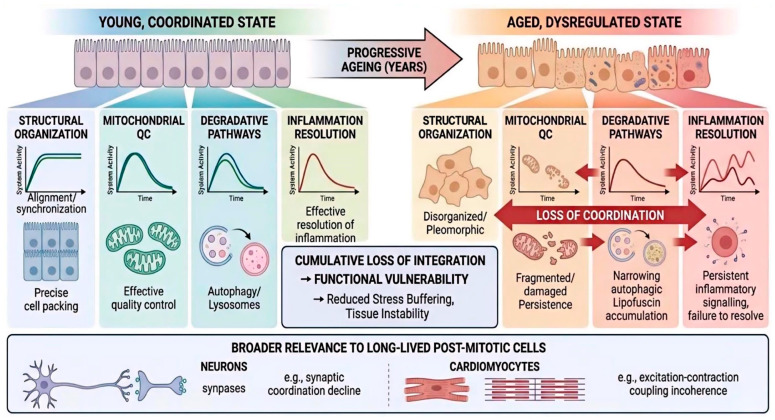
Retinal pigment epithelium ageing as a model of declining coordination among homeostatic systems in long-lived post-mitotic cells. This integrative model places RPE ageing within a broader framework of cellular ageing in long-lived post-mitotic tissues. In the ageing RPE, structural organisation becomes less precise, mitochondrial activity persists despite impaired quality control, degradative pathways operate with narrowing reserve, and inflammatory signalling remains engaged while resolution capacity declines. These systems do not fail simultaneously or disappear abruptly; rather, they remain active but progressively lose connectivity. Functional vulnerability emerges from this cumulative loss of integration, reducing the ability of cells to buffer additional stress and maintain tissue stability over time. The figure also highlights the broader relevance of this framework to other long-lived cell types, including neurons and cardiomyocytes, where ageing may likewise reflect declining coherence among adaptive systems rather than simple loss of capacity. QC, quality control.

Taken together, these observations support a system-level interpretation. In the ageing RPE, structure, metabolism, proteostasis, and inflammation remain active, but become progressively less well aligned. Functional vulnerability emerges from loss of coordination among these interacting systems rather than abrupt collapse of any single one. This view does not replace existing ageing frameworks; rather, it integrates them within a context where long-term maintenance, not rapid turnover, is the dominant biological challenge. This system-level interpretation is illustrated in Figure 4, which emphasises that ageing in the RPE reflects declining concert among still-active homeostatic systems rather than abrupt collapse of any single mechanism.

## 7. Outstanding Questions and Conclusions

Important questions remain, but the main challenge is no longer a lack of descriptive detail. Rather, it is the need to define the temporal and causal architecture of RPE ageing with greater precision. Current evidence has established that structural remodelling, mitochondrial dysfunction, lysosomal and proteostatic burden, and chronic inflammatory dysregulation are all central features of the ageing RPE. What remains less clear is how these processes are ordered, how they interact across time, and which of them primarily drive the transition from compensated ageing to functional decline. This limitation reflects a broader problem in ageing research: cross-sectional observations and experimentally compressed stress models are highly informative, yet they cannot fully capture the slow, cumulative, and interconnected nature of change in long-lived post-mitotic tissues. Future progress will therefore depend on longitudinal and integrative approaches that can resolve ageing not as a collection of endpoints, but as a trajectory.

This issue is especially important in the RPE because the tissue does not age under conditions of passive quiescence. It persists for decades under continuous oxidative, metabolic, and phagocytic load, while maintaining a level of structural and functional precision that is essential for retinal homeostasis. In that context, categories borrowed from proliferative tissues require particular caution. Senescence-like features, stress-response programmes, and survival-associated remodelling cannot be assumed to represent purely terminal dysfunction. Some may instead reflect transient stabilising states that preserve epithelial integrity under chronic burden, even as they narrow the tissue’s long-term adaptive reserve. Distinguishing maladaptive deterioration from compensatory persistence will be essential, both conceptually and therapeutically. Without that distinction, interventions directed at removing apparently dysfunctional cells or suppressing prominent downstream phenotypes may oversimplify the biology they aim to correct.

At the same time, these uncertainties do not weaken the framework that has emerged from the current work. They sharpen it. The most consistent insight across the field is that RPE ageing is not best understood as abrupt failure of a single pathway, nor as the additive accumulation of unrelated lesions. It is better viewed as a declining homeostatic coordination across the systems that sustain long-term cellular homeostasis. Structural polarity becomes less exact, mitochondrial activity is maintained under diminishing quality control, degradative pathways continue to function with reduced flexibility, and inflammatory signalling becomes more persistent while less effectively resolved. The central problem, in other words, is not damage alone, but declining integration. Ageing in the RPE reflects the gradual erosion of the organisational logic that allows a long-lived epithelial cell to buffer stress, maintain polarity, process burden, and remain functionally coupled to the neural retina over time.

This perspective has implications that extend beyond retinal biology. As a highly specialised, long-lived, post-mitotic epithelium exposed to lifelong physiological stress, the RPE offers a particularly informative system in which to examine how ageing unfolds when survival is prolonged but resilience progressively contracts. It provides a model for understanding how cells can remain present, yet become less precise; how homeostatic systems can remain active, yet become less coordinated; and how chronic dysfunction can emerge without immediate or widespread cell loss. For that reason, the RPE is valuable not only as a site of age-related retinal disease, but also as a broader framework for thinking about ageing in long-lived cellular systems.

The translational implication is equally important. Therapeutic success is unlikely to come from optimising a single protective pathway while leaving the wider network of homeostatic relationships unresolved. More effective strategies may need to restore coherence across metabolic control, organelle quality surveillance, proteostatic capacity, structural organisation, and inflammatory restraint. Framed in this way, the ageing RPE does not simply illustrate a tissue-specific degenerative process. It defines a broader biological problem: how long-lived cells preserve function across decades, and why they ultimately fail when the integration of their maintenance systems can no longer be sustained.

## Figures and Tables

**Table 1:** Representative structural, molecular, and functional changes in the ageing retinal pigment epithelium.

Change	Mechanistic Basis	Functional Consequence	Key References
Increased cell size, shape variability, and reduced hexagonal packing	Remodelling of polarity, cytoskeletal tension, and cell–cell adhesion	Reduced epithelial precision and stability despite preserved monolayer continuity	[11,12,13,14]
Regional heterogeneity of ageing changes	Differences in local metabolic demand and microenvironmental stress	Uneven vulnerability across macular, equatorial, and peripheral RPE	[13,14,58,59]
Bruch’s membrane thickening and extracellular matrix alteration	Matrix remodelling and lipid-rich deposit accumulation	Increased diffusional and mechanical constraint on RPE support	[17,18]
Declining efficiency of photoreceptor outer segment processing	Lifelong heterophagic load with reduced lysosomal reserve	Greater burden of incompletely degraded material	[27,28,29,30,31,32,33]
Lipofuscin accumulation and lysosomal stress	Incomplete degradation of outer segment-derived and autophagocytosed material	Impaired clearance and heightened susceptibility to cellular injury	[28,29,30,31,32]
Mitochondrial heterogeneity and impaired quality control	Altered fission–fusion balance, reduced mitophagy, disturbed organelle turnover	Less predictable metabolic output and increased oxidative stress	[19,20,21,22,26,27]
Persistent metabolic activity with dysregulated nutrient sensing	Altered oxidative phosphorylation and sustained mTORC1 signalling	Reduced regulatory precision and maladaptive anabolic persistence	[23,24,25]
Reduced proteostatic reserve	Decline in autophagic, lysosomal, and proteasomal efficiency	Accumulation of damaged proteins and organelles	[25,33,34,35]
Persistent low-grade inflammatory activation	Complement-related signalling, cytokine responses, and impaired resolution	Chronic inflammatory tone amplifies tissue vulnerability	[38,39,40,41,42,43,44,45]
Senescence-associated phenotypes and functional drift	Mitochondrial dysfunction, inflammatory reprogramming, and impaired stress adaptation	Viable but functionally altered cells persist before overt cell loss	[50,51,52,53,54,55,56,57,60,61]

Representative findings discussed in this review indicate that ageing in the RPE is expressed less as abrupt failure of a single pathway than as loss of system coherence among structural, metabolic, proteostatic, and inflammatory homeostatic systems.

## Data Availability

No new data were created or analysed in this study. Data sharing is not applicable to this article.

## Abbreviations

The following abbreviations are used in this manuscript:

A2E	N-retinylidene-N-retinylethanolamine
AMD	Age-related macular degeneration
ATP	Adenosine triphosphate
DRP1	Dynamin-related protein 1
mTORC1	Mechanistic target of rapamycin complex 1
PGAM5	Phosphoglycerate mutase family member 5
p16^INK4a	Cyclin-dependent kinase inhibitor 2A
p21^CIP1	Cyclin-dependent kinase inhibitor 1A
RPE	Retinal pigment epithelium
ROS	Reactive oxygen species
SA-β-gal	Senescence-associated β-galactosidase
SUMO	Small ubiquitin-like modifier
